# A donor-specific QTL, exhibiting allelic variation for leaf sheath hairiness in a nested association mapping population, is located on barley chromosome 4H

**DOI:** 10.1371/journal.pone.0189446

**Published:** 2017-12-07

**Authors:** Stephanie Saade, Burcu Kutlu, Vera Draba, Karin Förster, Erika Schumann, Mark Tester, Klaus Pillen, Andreas Maurer

**Affiliations:** 1 King Abdullah University of Science and Technology (KAUST), Biological and Environmental Sciences and Engineering (BESE), Thuwal, Saudi Arabia; 2 Ege University, Department of Biotechnology, Erzene, Bornova/İzmir, Turkey; 3 Institute of Agricultural and Nutritional Sciences, Department of Plant Breeding, Martin Luther University Halle-Wittenberg, Halle, Germany; 4 Institute of Agricultural and Nutritional Sciences, Department of Agronomy and Organic Farming, Martin Luther University Halle-Wittenberg, Halle, Germany; Julius Kühn-Institut, GERMANY

## Abstract

Leaf sheath hairiness is a morphological trait associated with various advantages, including tolerance to both abiotic and biotic stresses, thereby increasing yield. Understanding the genetic basis of this trait in barley can therefore improve the agronomic performance of this economically important crop. We scored leaf sheath hairiness in a two-year field trial in 1,420 BC_1_S_3_ lines from the wild barley nested association mapping (NAM) population HEB-25. Leaf sheath hairiness segregated in six out of 25 families with the reference parent Barke being glabrous. We detected the major hairy leaf sheath locus *Hs* (syn. *Hsh*) on chromosome 4H (111.3 cM) with high precision. The effects of the locus varied across the six different wild barley donors, with donor of HEB family 11 conferring the highest score of leaf sheath hairiness. Due to the high mapping resolution present in HEB-25, we were able to discuss physically linked pentatricopeptide repeat genes and subtilisin-like proteases as potential candidate genes underlying this locus. In this study, we proved that HEB-25 provides an appropriate tool to further understand the genetic control of leaf sheath hairiness in barley. Furthermore, our work represents a perfect starting position to clone the gene responsible for the 4H locus observed.

## Introduction

Trichomes are specialized plant surface structures including hairs and exudate-secreting glands that originate and extend from the epidermis layer of aerial tissue [1]. They vary greatly in morphology, cellular composition, density, location, and function [1,2]. Non-glandular trichomes, through their reflectance properties, could play a role in temperature regulation of transpiring surfaces and the reduction of the heat load on leaves by reducing the absorption of radiant energy [3,4]. Interestingly, the wheat cultivar Pak 15800, which produced the largest number of leaf hair under water stress, was ranked as the most drought tolerant wheat accession in Hameed et al. [5]. Hence, hairiness could be a good selection criterion for drought tolerance, at least in wheat as well as barley as a temperate cereal [5]. Furthermore, hairiness could act as a deterrent to certain biotic stresses [6]. For instance, Kim et al. [7] reported a close association between trichome density and pepper mottle virus resistance in pepper, where the virus resistance could be due to the deterrence of the insect vectors of the virus by the hairs.

A number of studies on the molecular mechanism of the formation of trichomes have been conducted in Arabidopsis. Positive regulators of trichome formation include GLABROUS1 (GL1), an R2R3 MYB transcription factor; GLABRA3 (GL3), a basic helix-loop-helix transcription factor; Transparent Testa Glabra 1 (TTG1), a WD40 protein; and GLABRA2 (GL2), a homeodomain leucine zipper transcription factor. Triptychon (TYR) is an R3 MYB transcription factor and a negative regulator of trichome formation [8,9]. GL1 interacts with GL3, which interacts with TTG1. The GL1-GL3-TTG1 complex binds to the promotor of *GL2* to initiate *GL2* expression [10]. Epidermal cells expressing the GL2 protein are able to develop into trichome cells [9]. The GL1-GL3-TTG1 complex also binds to the promoter of *TYR*. The TYR protein expressed in trichome cells can move into neighboring cells and compete with GL1 for binding to GL3 to repress the *GL2* expression [10].

In barley, leaf sheath hairiness is thought to follow a dominant single gene mode of inheritance [11,12]. The *Hairy leaf sheath* locus *(Hs* syn. *Hsh)* has initially been discovered by Pickering et al. [13]. The locus *Hs*_*b*_ is derived from *Hordeum bulbosum* and is allelic to *Hs* [13]. Furthermore, it is homologous to *Hairy peduncle Hp1*, which governs leaf sheath and peduncle hairiness in rye and maps to the long arm of chromosome 5 [14]. The collinearity of the 5L rye chromosome to the distal part of the 4L chromosomes of other *Triticeae* members is due to a translocation event [15]. In addition, *Hp1* and *Hs*_*b*_ line up with *Hairy leaf 1* (*Hl1)* (chromosome 4BL) that governs leaf hairiness in wheat [16]. Recently, Wang et al. [17] mapped a QTL controlling leaf sheath hairiness on chromosome 4HL in an association mapping diversity panel of 569 barley cultivars using 1,042 SNP markers from the Illumina GoldenGate oligonucleotide pool assay 1 (BOPA1). Furthermore, Wan et al. [18] detected a strong QTL on chromosome 4D associated with leaf sheath hairiness in wheat. The wild allele at this QTL originated from *Aegilops tauschii*, *a wild* progenitor of the hexaploid wheat, and was significantly associated with increased grain yield, grain weight and grain weight per spike [18]. The authors suggested that DNA markers for leaf sheath hairiness could be used for marker-assisted selection to increase yield in wheat [18].

In the present study, we used ‘Halle Exotic Barley 25’ (HEB-25), a recently developed nested association mapping (NAM) population, to study biodiversity of leaf sheath hairiness in wild barley. HEB-25 was developed by crossing 25 wild barley (*Hordeum vulgare* ssp. *spontaneum*, *Hsp*, and ssp. *agriocrithon*, *Hag*) accessions with the spring barley cultivar Barke (*H*. *v*. ssp. *vulgare*, *Hv*), ultimately resulting in 1,420 HEB lines in BC_1_S_3_ generation [19]. The fact that six wild HEB donors exhibit hairy leaf sheaths whereas Barke is glabrous makes HEB-25 an excellent tool (1) to identify QTL controlling hairiness in barley, (2) to locate the respective QTL at an increased genetic resolution and (3) to characterize the allelic variation at the respective QTL.

## Materials and methods

### Plant material

In this study, 1,420 HEB lines from HEB-25 were grown in the field. HEB-25 is a barley NAM population resulting from crosses between the two-rowed German spring barley cultivar Barke and 25 wild donors. One of the wild donors is a Tibetan *H*. *vulgare* ssp. *agriocrithon* (*Hag*) accession and the other 24 accessions are *H*. *vulgare* ssp. *spontaneum* (*Hsp*) originating from the Fertile Crescent [19]. F_1_ plants were backcrossed to Barke and selfed three times through single seed descent. The lines used in this study were in BC_1_S_3:6_ in the field trial of 2013 and in BC_1_S_3:8_ in the field trial of 2015. Further details about the development of HEB-25 are available in Maurer et al. [19].

### Field trials

A two-year field trial (2013 and 2015) was conducted at the ‘Kühnfeld Experimental Station’ of the Martin Luther University, Halle-Wittenberg (51°29′46.47″N; 11°59′41.81″E). A complete randomized block design was applied, where the 1,420 HEB lines were grown in two replicates (two blocks) per year. In 2013, the plots consisted of two rows (30 seeds each) with a length of 1.50 m, a distance of 0.20 m between rows and a spacing of 0.50 m between plots. In 2015, plots dimensions were identical, however sowing density was increased to 50 seeds per row. Barke was integrated as a check line in all trials. All field trials were sown in March (i.e. early spring), with fertilization and pest management carried out according to local practice.

### Phenotyping of HEB-25

Hairiness of the leaf sheath was recorded after plants reached shooting stage (BBCH31 stage) and before flowering (BBCH49 stage, recorded as the number of days from sowing until 50% of the plants in the plot had their first awns visible) [20]. Fertilization occurs at this stage of spring barley development [21]. Based on visual assessment, the density of hairs covering the leaf sheath was scored. Therefore, a scale of 1–7 was used, where “1” is the score for glabrous (non-hairy) leaf sheaths, and “3”, “5” and “7” are the scores for leaf sheaths that are slightly hairy, medium hairy and very hairy, respectively. Photographs illustrating each hairiness score are given in S1 Fig. Detailed microscopic images were created for Barke and all 25 wild barley donors as well as for all lines of HEB family 23 using a VHX-500 F digital 3D microscope (Keyence, Osaka, Japan).

### Genotyping of HEB-25

HEB-25 lines, Barke, and the 25 donors were genotyped with 7,864 SNPs using the barley Infinium iSelect 9k SNP chip [22]. In total, 5,709 SNPs, which were polymorphic in at least one HEB family, showed less than 10% missing data and less than 12.5% heterozygosity, were used for subsequent analyses. Based on parental genotype information, the exotic allele could be specified in each segregating family, where homozygous Barke genotypes, heterozygous genotypes and homozygous exotic genotypes were assigned scores of 0, 1 and 2 at each SNP locus, respectively. Score values, thus, represent the number of wild barley alleles at each SNP (genotype data is available in Additional file 5 in Maurer et al. [19]).

### Phenotypic data analysis

We used SAS 9.4 software (SAS Institute Inc., Cary, NC, USA) to analyze the phenotypic data. Pearson correlation was used to show association of leaf sheath hairiness between years. A mixed linear model was fitted to calculate genotype, year and genotype-by-year interaction effects. These effects were assumed random to estimate variance components and determine broad-sense heritability (h^2^) across the whole population based on the entry means.

${\text{h}}^{2}=\frac{\sigma_{\text{G}}^{2}}{\sigma_{\text{G}}^{2}+\frac{\sigma_{\text{GY}}^{2}}{\text{y}}+\frac{\sigma_{\text{R}}^{2}}{\text{yr}}}$, where $\sigma_{\text{G}}^{2}$, $\sigma_{\text{GY}}^{2}$, and $\sigma_{\text{R}}^{2}$ represent the genotypic, genotype-by-year, and residual variance components; while y and r refer to the number of years and the number of replicates, respectively.

The genotype effect was considered fixed to derive the best linear unbiased estimates (BLUEs). BLUEs were used for the subsequent genome-wide association study (GWAS).

### Association mapping analysis

We conducted a GWAS on the BLUEs of the trait using Model-A, as suggested in Liu et al. [23], where cofactors were included in addition to the tested SNP in the multiple linear regression model. Würschum et al. [24] showed that this model revealed the highest predictive power and detected the highest number of QTL in joint linkage association mapping. This model allows the post-hoc determination of family-specific QTL effects [25], which is explained further down. The analysis was conducted in SAS and consisted of two steps. In the first step, cofactors, i.e. SNPs controlling population structure and genetic background, were selected by stepwise forward-backward regression. Overfitting was prevented by minimizing the Schwarz Bayesian Criterion (SBC or BIC) [26]. In the second step, an *F* test compared a reduced model (without the tested SNP effect) with a full model (with the tested SNP effect included) for each of the 5,709 SNPs to identify SNPs associated with the phenotypic trait [27]. Resulting p-values were adjusted by Bonferroni–Holm procedure to reduce false positives resulting from multiple testing [28]. Only SNPs having an adjusted p-value <0.05 were considered significant. The R package qqman [29] was used to create the Manhattan plot. The amount of phenotypic variance explained by a single significant SNP, R^2^, was estimated by fitting only that SNP in a linear model and R^2^_adj_ of all significant SNPs together was calculated according to Utz et al. [30]. In addition, prediction ability (R^2^_val_) was determined in a five-fold cross-validation scenario. For this, 100 subsets were extracted out of the total phenotypic data. Each subset consisted of 80% randomly chosen HEB lines per family. This set was used as the training set to define significant markers and to estimate their effects, while the remaining 20% of lines were used as the validation set. Thereby the phenotypes of the validation set lines (20%) were predicted based on marker effects estimated in the training set (80%). Prediction ability (R^2^_val_) was then calculated as the squared Pearson product-moment correlation between the observed and predicted phenotypes of the validation set. The average of all 100 runs was then taken as the final record for R^2^_val_.

The effect of the wild allele of a significant SNP relative to the Barke allele was calculated by multiplying the regression coefficient (α effect) of that SNP by a factor of two, meaning the replacement of two Barke alleles with two wild barley alleles.

To estimate a family-specific QTL effect we cumulated significant SNP marker effects as described in Maurer et al. [25]. First, a peak marker for each expected donor-specific QTL was selected where the p-value was minimized. Each peak marker was placed centrally in a 26 cM interval (reflecting the mean introgression size in HEB-25) to look for further significant SNPs in this region. Effect estimates of all markers within this interval were then cumulated for each of the 25 donors. Since SNPs show different identity-by-state segregation patterns across the donors of HEB families, a different cumulated effect was obtained for each donor. In contrast to a haplotype-based approach, this method is more phenotypically orientated and is able to group family effects based on phenotypically-relevant SNP effects.

In order to identify potential candidate genes underlying the significant peak markers, we used the BARLEYMAP pipeline [31] with the recently released barley sequence [32]. We looked for genes within a window of 3 cM from the peak marker SCRI_RS_107762 and discussed the ones with a potential role in trichome formation and development based on literature review.

## Results and discussion

### Phenotypic data analysis

We phenotyped leaf sheath hairiness of 1,420 lines belonging to HEB-25, a wild barley nested association mapping population (S1 Table). Phenotyping was carried out for two years and the Pearson correlation between the years is equal to r = 0.82. The correlation is high, although lower than expected, possibly because the trait was recorded quantitatively (a score on a scale from 1–7) rather than scoring presence/absence. Also, different persons carried out the visual scoring in 2013 (BK) and 2015 (SS), adding another source of variation between years. Nevertheless, leaf sheath hairiness was found to be highly heritable with h^2^ = 80.4%, where the phenotypic variance in HEB-25 is predominantly controlled by genetic effects (Table 1).

**Table 1:** Variance partitioning and heritability of leaf sheath hairiness in HEB-25.

Trait	Genotype	Year	Genotype-by-year	Residual	Broad-sense heritability (%)
Leaf sheath hairiness	0.559	0.014	0.248	0.050	80.4

**Fig 1:**
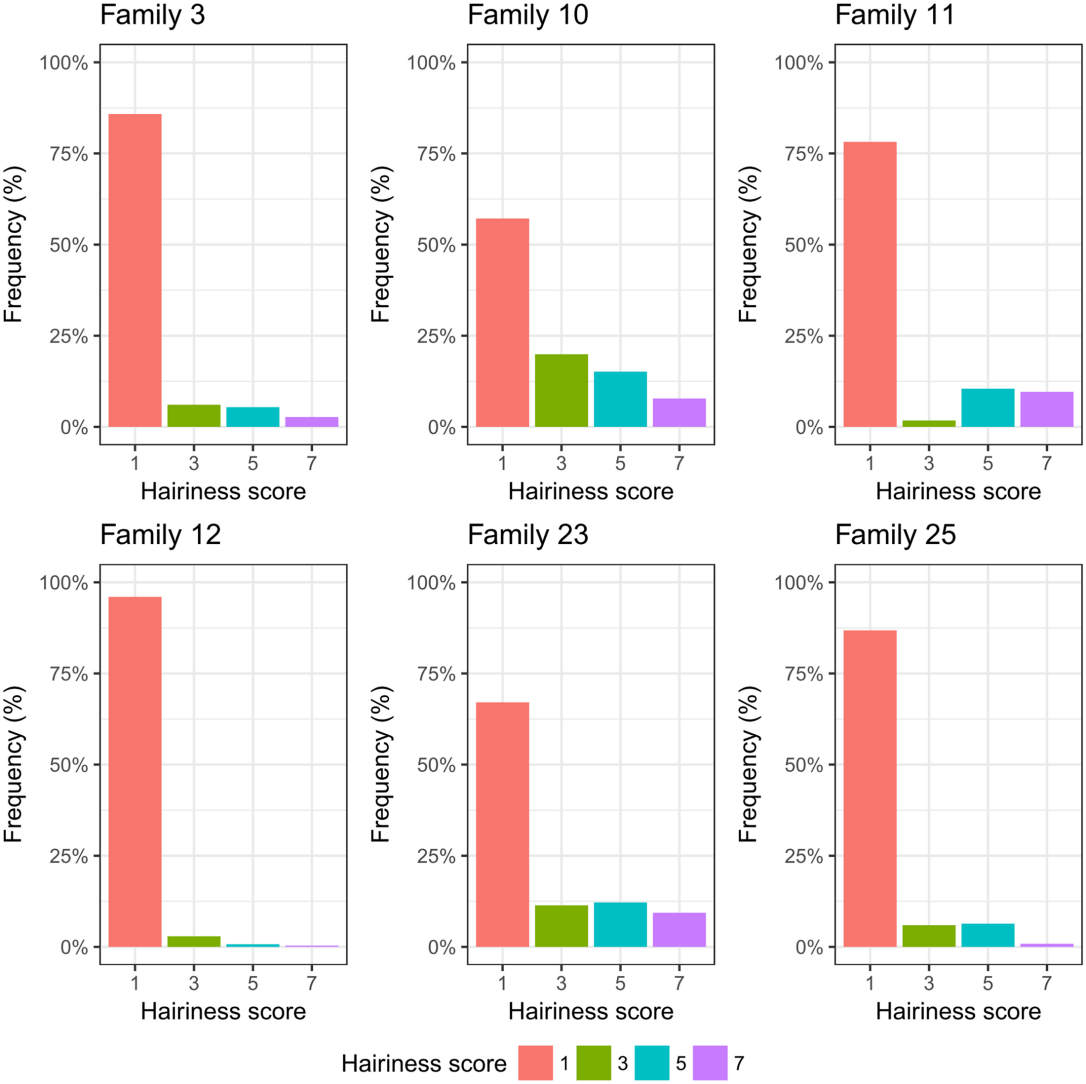
Frequency distribution (in %) of leaf sheath hairiness scores for six segregating HEB families.

Out of 25 HEB families tested, leaf sheath hairiness segregated in six families (Table 2). Frequency distributions of the hairiness scores for each of the six hairiness HEB families are illustrated in Fig 1. In each family, the majority of HEB lines (>55%) revealed a non-hairy phenotype. The highest frequencies of hairy phenotypes were found in HEB families 10, 11 and 23. The observed hairness segregation suggests a monogenic or oligogenic inheritance for leaf sheath hairiness in the respective six segragting HEB families. The geographic origin of the HEB-25 wild barley donors are pinpointed in a map shown in S2 Fig. It occurs that most hairy donors (5 out of 6) originate from coastal regions of the Mediterranean Sea, whereas non-hairy donors predominantly originate from non-coastal regions of the Fertile Crescent. Possibly, there appears to be a selective advantage at coastal regions favoring the presence of leaf sheath hairiness, which might be associated with an increased coastal wind movement. In this regard, Grace and Russell [33] reported that the grass *Festuca arundinacea*, grown under windy field conditions, had more adaxial macro hairs than those grown in calm greenhouse conditions. In addition, we could postulate the association of leaf sheath hairiness with tolerance to higher temperatures since coastal regions tend to have lower elevations and higher temperatures than other regions.

**Table 2:** Descriptive statistics for six HEB families segregating for leaf sheath hairiness.

HEB family (n) ^(a)^	Wild donor	Origin of donor	Lines with score 7 (%)^(b)^	Mean^(c)^	Min^(d)^	Max^(d)^	SD ^(e)^	CV (in %) ^(f)^
3 (296)	HID_055	Turkey	2.7	1.50	1	7	1.36	90.36
10 (231)	HID_102	Syria	7.8	2.47	1	7	1.97	79.65
11 (229)	HID_109	Syria	9.6	2.03	1	7	2.04	100.46
12 (276)	HID_114	Lebanon	0.4	1.11	1	7	0.59	53.50
23 (246)	HID_359	Israel	9.3	2.28	1	7	2.04	89.60
25 (235)	HID_386	Israel	0.9	1.43	1	7	1.18	82.73

The 19 HEB families, not displayed in this table, did not reveal leaf sheath hairiness phenotypes.

^(a)^ HEB family and number of observations (n) for the hairiness score.

^(b)^ Percentage of lines (from n) with a leaf sheath hairiness score of 7 within each family.

^(c)^ Mean of all lines of the respective family.

^(d)^ Minimum and maximum scores for hairiness, where “1” is the score for glabrous (non-hairy) leaf sheaths and “7” is the score for leaf sheaths that are very hairy.

^(e)^ Standard deviation.

^(f)^ Coefficient of variation.

When examined under the microscope, the hairs present in HEB-25 lines are enlarged at the base and pointy at the tip (S3 Fig). Further studies are needed to investigate hair length and density as well as to identify the exact morphology of these hairs including whether they are unicellular or multicellular. Interestingly, all 63 HEB lines investigated under the microscope, as well as all the 25 wild donors and Barke, carried a second type of hair (designated with the letter B in S3 Fig), in addition to the macroscopically visible type that is scored in this study. These additional hairs are microscopic and thorn-like (S3 Fig). Further molecular and genetic investigations are needed to elucidate a potential role of these thorn-like hairs in biotic and abiotic tolerance.

### Association mapping analysis

**Fig 2:**
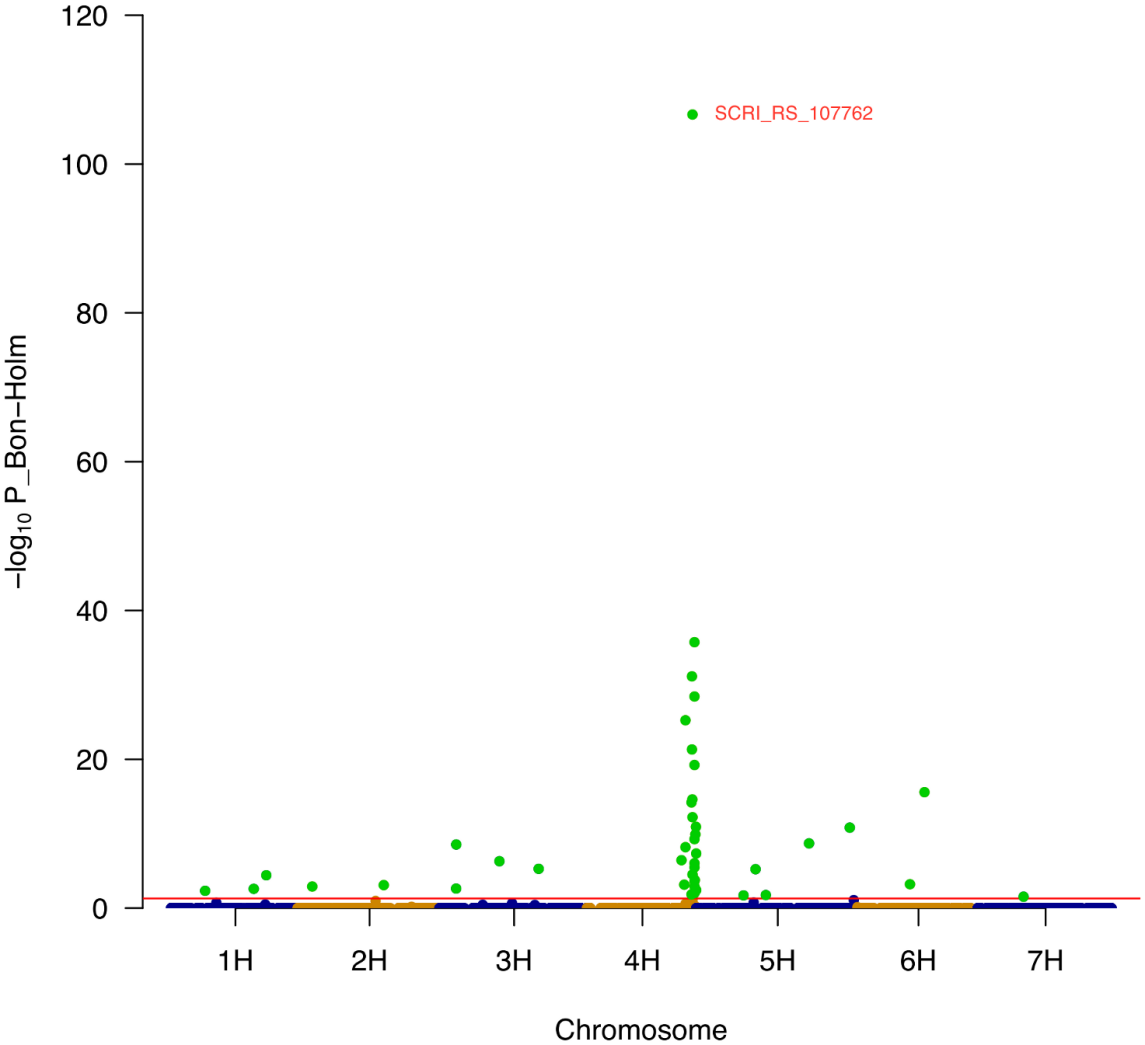
Manhattan plot for GWAS analysis of leaf sheath hairiness. The y-axis shows the −log_10_ of Bonferroni-Holm-adjusted P-values. The horizontal red line (−log_10_ P_Bon-Holm = 1.3) indicates the significance threshhold of P_Bonferroni-Holm = 0.05. Green dots indicate signifcant SNPs exhibiting a Bonferroni-Holm corrected p-value <0.05.

Based on the phenotypic data, we carried out a genome-wide association study on barley leaf sheath hairiness using 5,709 polymorphic SNP markers from the Infinium iSelect 9k SNP chip. In total, 44 significantly associated SNPs with P_Bonferroni-Holm<0.05 were detected (Table 3 and S2 Table). These SNPs were confounded to 16 QTL regions, present on all seven barley chromosomes (Fig 2 and S3 Table). The explained phenotypic variation (R^2^_adj_), calculated across all 44 significant SNPs, was equal to 0.84 and prediction ability (R^2^_val_) was 0.72 after cross-validation (Table 3). These findings indicate that the model used was able to explain an exceptionally large proportion of the phenotypic variation of leaf sheath hairiness in HEB-25.

**Table 3:** Summary of association mapping results for leaf sheath hairiness.

Trait	Number of significant markers	R^2^_adj_^(a)^	R^2^_val_^(b)^
Leaf sheath hairiness	44	0.84	0.72

^(a)^ Explained phenotypic variation based on linear regression of all 44 significant markers.

^(b)^ Prediction ability obtained as an average of predicting 20% of lines in 100 cross-validation runs.

**Fig 3:**
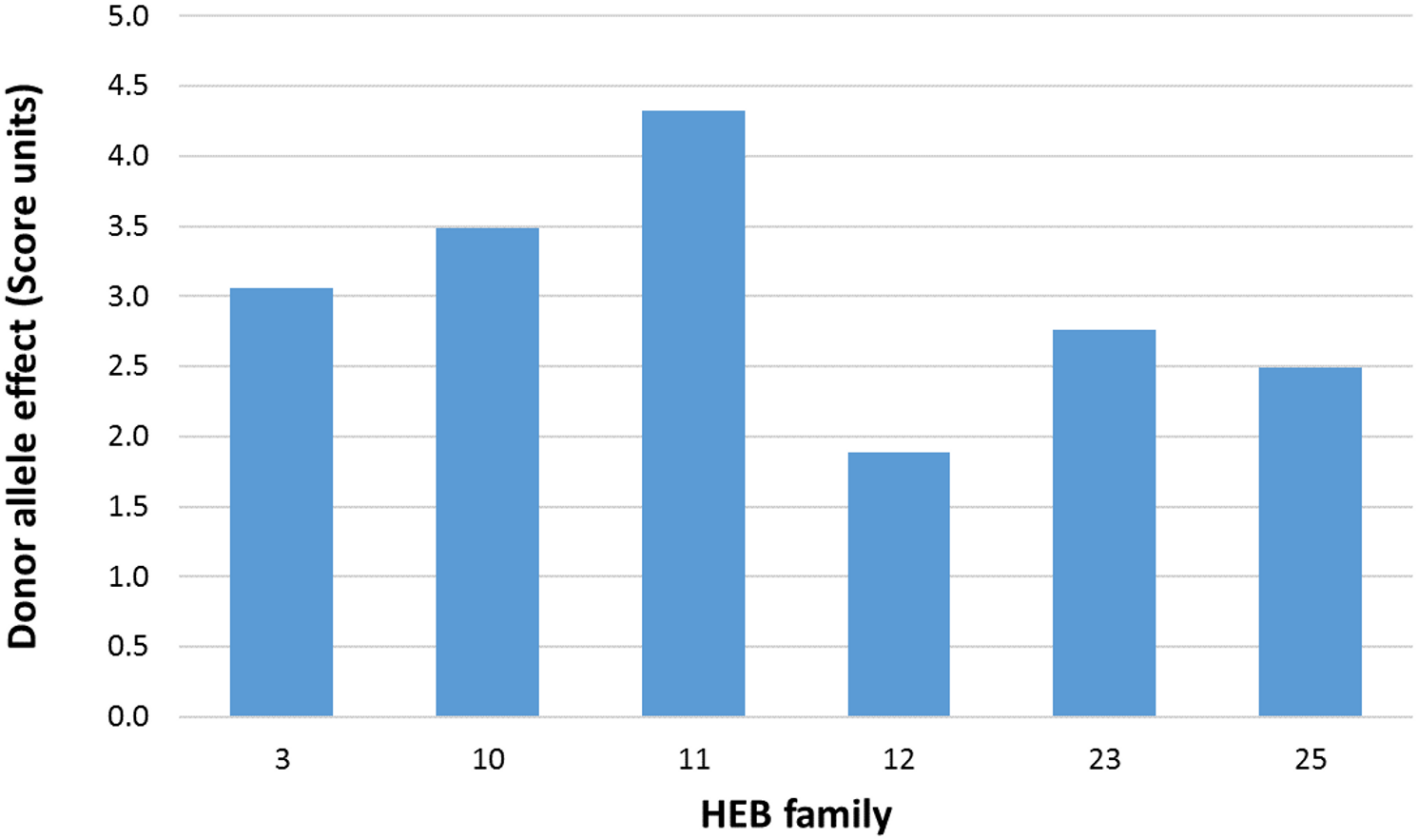
Estimation of cumulated wild barley donor-specific allele effects at the major QTL on chromosome 4H (at 111.3 cM) for six leaf sheath hairness HEB families.

Among the 16 QTL, we detected a major QTL on chromosome 4H with high precision at position 111–113 cM. The most significant SNP, SCRI_RS_107762 at position 111.3 cM, revealed the highest Bonferroni-Holm adjusted -log_10_ P value of 107. The wild barley allele at this SNP had an increasing effect on leaf sheath hairiness equal to 2.54 score units relative to the Barke allele; in other words, the wild allele at this SNP increased leaf sheath hairiness score by 2.54 in comparison to the Barke leaf sheath hairiness score of 1. Clustering of a high number of 27 significant markers in this genomic region suggests the presence of family-specific effects [25]. Therefore, we calculated the cumulated wild barley donor-specific allele effect at the major QTL on chromosome 4H separately for each HEB family (Fig 3 and S3 Table). We found contrasting donor-specific effects ranging from 1.88 to 4.33 among the six polymorphic HEB families. The wild donor allele present in HEB family 11 conferred the highest effect on hairiness, where each line carrying a homozygous wild barley genotype is expected to have a hairiness score increased by 4.33 units compared to those lines carrying the homozygous Barke genotype.

We used the BARLEYMAP pipeline [31] with the recently released barley sequence [32] to identify potential candidate genes, within a window of 3 cM from the peak marker SCRI_RS_107762, to explain the QTL. Here, we discuss the genes that might be candidates for leaf sheath hairiness based on their function and on literature reports linking the genes to hairiness. In the QTL region 4H-111.3 cM, pentatricopeptide repeat genes (*HORVU4Hr1G085920*.*1*) are present.

In plants, pentatricopeptide repeat (PPR) proteins constitute a large family of proteins characterized by a degenerate 35 amino acids motif in tandem repeats [34]. A large portion of these genes are targeted to mitochondria and chloroplasts [35] and are involved in organellar RNA processing [36–38]. Their roles also include seed development and growth [39], restoration of fertility in cytoplasmic male sterile plants[40], and response to abiotic stresses [41].

Interestingly, members of these gene families were found by Zeng et al. [42] when they fine mapped the *glabrous leaf 6 (GL6)* hairiness gene of *Oryza sativa*. In addition, Spyropoulou et al. [43] identified pentatricopeptides among the 30 most common InterPro entries in the RNAseq analysis of the *Solanum lycopersicum* trichomes.

Another candidate gene in that region is a subtilisin-like protease (*HORVU4Hr1G085590*). In fact, stomatal density and distribution 1–1 (SDD1) and abnormal leaf shape1 (ALE1) are two subtilisin-like proteases that were shown to be involved in the differentiation of epidermal cells in *Arabidopsis*: SDD1 protease is required in stomatal cell formation [44,45], while ALE1 plays a role in cuticle formation [46]. Furthermore, Liu et al. [47] and Luo et al. [48] showed that transgenic tobacco overexpressing *Solaneum americanum SaPIN2b* and *SaPIN2a*, which are serine protein inhibitors with *SDD1* and *ALE1* as possible targets, had significantly higher trichome density and increased branching. In addition, jasmonic acid-insensitive 1–1 (*jai1-1*) tomato mutants, which cannot produce PIN2, had significanly fewer glandular trichomes than wild type plants [49].

**Fig 4:**
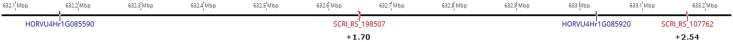
Physical map of the QTL region 4H-111.3cM including both candidate genes. The position of the two most significant SNP markers is indicated as well as their wild allele effects on the hairiness score.

Both candidates lie within a 1 Mb interval in close proximity to the most significant SNP markers of the QTL region 4H-111.3 cM (Fig 4).

Patterson [12] and Taketa and Takeda [11] described leaf sheath hairiness in barley as a monogenic trait and also Cockram et al. [50] specified that hairiness of leaf sheath is associated with a single locus. However, Wang et al. [17] and our study revealed additional minor QTL besides the major 4H QTL (Fig 2 and S2 Table). The genes underlying these additional QTL could be involved in fine tuning rather than in initiation of the development of epidermal hairs.

## Conclusions

This work showed that that the cumulation method [25] reliably identified QTL effects of hairy donors and that allelic series seem to exist in HEB-25. HEB-25 can, thus, be used as a tool for further analyses of leaf sheath hairiness in barley and for fine-mapping and, ultimately, cloning of the causative gene/s. In the future, functional characterization of hair development in HEB-25 and their potential to increase barley yields under harsh environmental conditions will be investigated. The high accuracy of QTL mapping due to the NAM population design and the Infinium iSelect 9k SNP chip already allowed narrowing down the position of the major QTL and defining potentially plausible candidate genes. As a strategy to clone the 4H-111.3 cM QTL in HEB-25 we propose to apply cloning by sequencing as outlined by Jost et al. [51]. For this, we will select HEB lines, which, in generation BC_1_S_3_, proved to be heterozygous at the most significant SNP, SCRI_RS_107762, and homozygous in the remaining genome background. After propagating these HEB lines to the current generation (BC_1_S_3:9_), two pools of sister lines could be selected, which are expected to be fixed in the genomic background and segregate 1:1 for leaf sheath hairiness. The resulting two sister pools will be used to select recombination events close to the SCRI_RS_107762 locus. Subsequently, these recombinants will be used for whole exome-capture resequencing to validate candidate genes [52]. The final identity of the true candidate gene, causing the 4H-111.3cM QTL effect on leaf sheath hairiness, could be achieved through a *CRISP/Cas9*-based targeted knock out of the candidate gene [53,54].

## Supporting information

S1 Fig Scores 1–7 used to characterize the leaf sheath hairiness phenotype found in HEB-25. The value of “1” refers to non-hairy leaf sheaths, whereas “3”, “5” and “7” indicate leaf sheaths, which are scored as slightly hairy, medium hairy, and very hairy, respectively.
(PDF)

S2 Fig Map indicating the geographic origin of wild barley donors of HEB-25. Red and white dots indicate origins of hairy and non-hairy donors of HEB-25, respectively.
(PDF)

S3 Fig Microscopic 3D image of a barley line revealing two types of leaf sheath hairiness. The elongated hair, designated with letter (A), is the type of hair segregating in HEB-25 and phenotyped in this study. The two thorn-like hairs, designated with letter (B), indicate a second type of hair, which was found in all investigated HEB lines and donors.
(PDF)

S1 Table a) Raw phenotypic data of leaf sheath hairiness in HEB-25 b) Best linear unbiased estimates (BLUEs) of leaf sheath hairiness in HEB-25. (XLSX)

S2 Table GWAS results of leaf sheath hairiness. (XLSX)

S3 Table Donor-specific QTL effects on leaf sheath hairiness for 25 HEB families. (XLSX)
