# Supplementary material for: A donor-specific QTL, exhibiting allelic variation for leaf sheath hairiness in a nested association mapping population, is located on barley chromosome 4H
Source: PLoS One. 2017 Dec 7;12(12):e0189446. doi: 10.1371/journal.pone.0189446 (PMC5720540; doi:10.1371/journal.pone.0189446)
Supplement: S1 Fig — The value of “1” refers to non-hairy leaf sheaths, whereas “3”, “5” and “7” indicate leaf sheaths, which are scored as slightly hairy, medium hairy, and very hairy, respectively. (PDF) [file pone.0189446.s001.pdf]

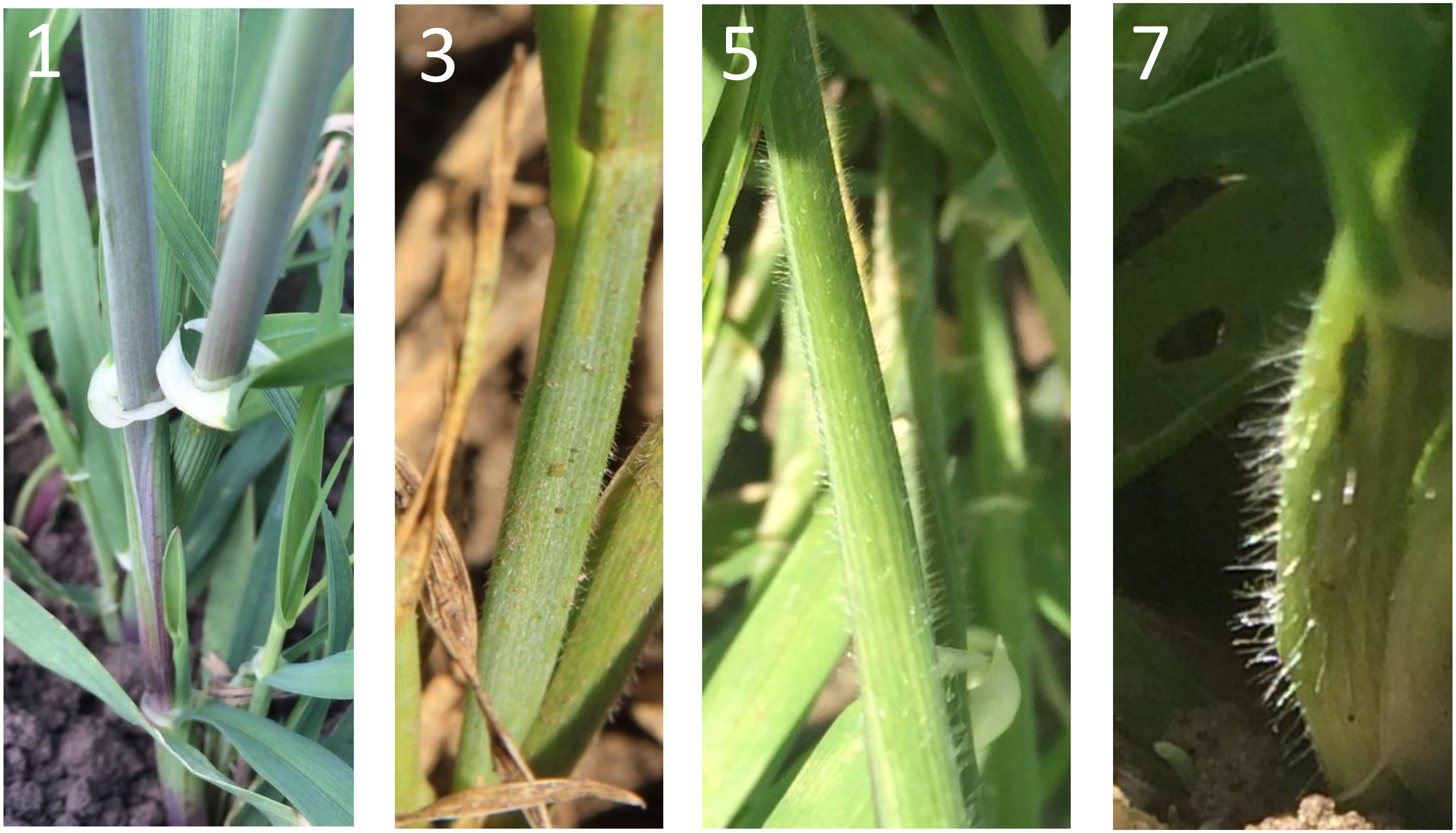

S1 Fig. Scores 1-7 used to characterize the leaf sheath hairiness phenotype found in HEB-25

The value of “1” refers to non-hairy leaf sheaths, whereas “3”, “5” and “7” indicate leaf sheaths, which are scored as slightly hairy, medium hairy, and very hairy, respectively.
