# Supplementary material for: A donor-specific QTL, exhibiting allelic variation for leaf sheath hairiness in a nested association mapping population, is located on barley chromosome 4H
Source: PLoS One. 2017 Dec 7;12(12):e0189446. doi: 10.1371/journal.pone.0189446 (PMC5720540; doi:10.1371/journal.pone.0189446)
Supplement: S2 Fig — Red and white dots indicate origins of hairy and non-hairy donors of HEB-25, respectively. (PDF) [file pone.0189446.s002.pdf]

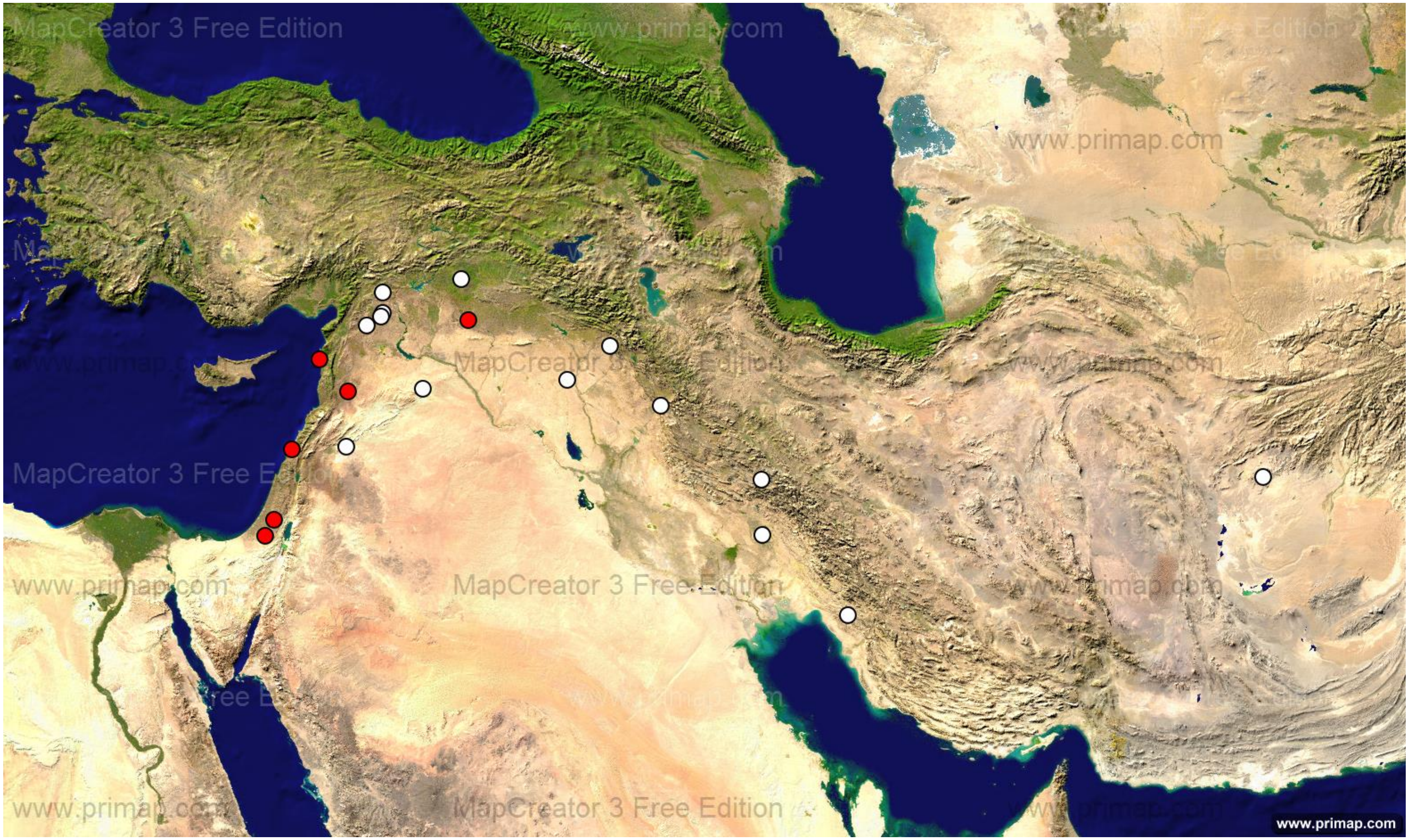

S2 Fig. Map indicating the geographic origin of wild barley donors of HEB-25

Red and white dots indicate origins of hairy and non-hairy donors of HEB-25, respectively.
