# Supplementary material for: A donor-specific QTL, exhibiting allelic variation for leaf sheath hairiness in a nested association mapping population, is located on barley chromosome 4H
Source: PLoS One. 2017 Dec 7;12(12):e0189446. doi: 10.1371/journal.pone.0189446 (PMC5720540; doi:10.1371/journal.pone.0189446)
Supplement: S3 Fig — The elongated hair, designated with letter (A), is the type of hair segregating in HEB-25 and phenotyped in this study. The two thorn-like hairs, designated with letter (B), indicate a second type of hair, which was found in all investigated HEB lines and donors. (PDF) [file pone.0189446.s003.pdf]

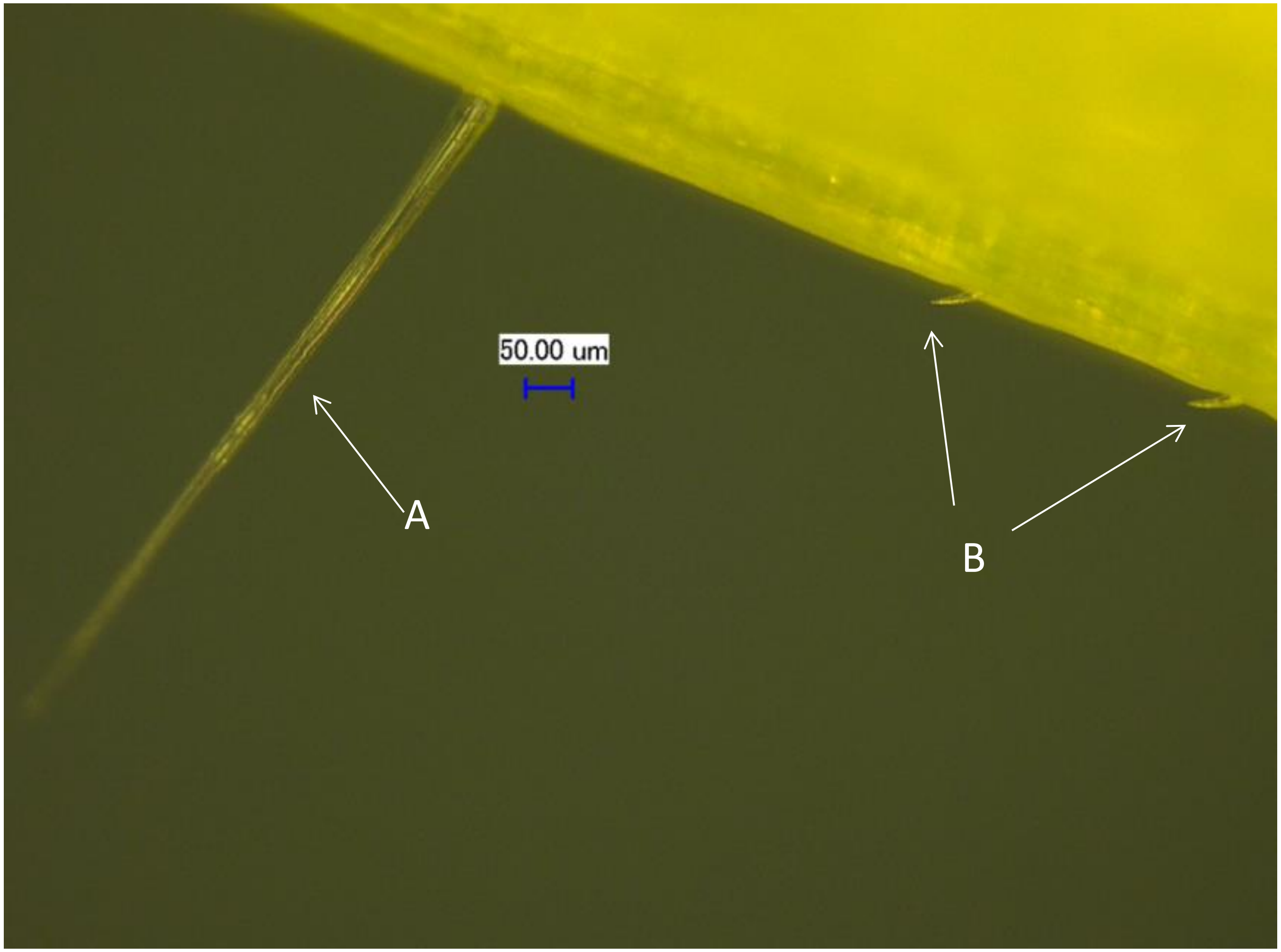

S3 Fig. Microscopic 3D image of a barley line revealing two types of leaf sheath hairiness

The elongated hair, designated with letter (A), is the type of hair segregating in HEB-25 and phenotyped in this study. The two thorn-like hairs, designated with letter (B), indicate a second type of hair, which was found in all investigated HEB lines and donors.
